# Effect of Environmentally Friendly Betalain Extraction Methods on Antioxidant Compounds of Tunisian *Opuntia stricta* Fruit

**DOI:** 10.3390/foods14050851

**Published:** 2025-03-01

**Authors:** Nadia Smirani, Souhir Bouazizi, Emna Bettaieb, Rachel Torkhani, Moktar Hamdi

**Affiliations:** 1 Laboratory of Microbial Ecology and Technology, National Institute of Applied Science and Technology (INSAT), University of Carthage, BP 676, Tunis 1080, Tunisia; nadia.smirani@fsb.ucar.tn (N.S.); souhir.bouazizi@insat.ucar.tn (S.B.); emna.bettaieb.ouni@gmail.com (E.B.); 2 Faculty of Sciences of Bizerte (FSB), University of Carthage, Zarzouna, Bizerte 7021, Tunisia; 3 National Institute of Research and Physico-Chemical Analysis (INRAP), Technopark of Sidi Thabet, Ariana 2020, Tunisia; torkhanirachel@yahoo.fr

**Keywords:** *Opuntia stricta*, betalains, red colorant, non-conventional extraction, microwave, ultrasound, bioactive antioxidant, drying

## Abstract

This study focuses on the extraction of betalain compounds from *Opuntia stricta* as a natural alternative to synthetic colorants and sustainable environmentally friendly technology solutions. Non-conventional extraction technologies including microwave (MW) and ultrasound (US) were used alone or in combination. The extraction process was conducted for both undried *Opuntia stricta* (OS) and dried *Opuntia stricta* (DOS) plant material at two distinct drying temperatures, 40 °C and 60 °C, to assess the stability of betalain molecules. The colorant’s potential was evaluated by determining the betalain content, total phenolic content, and antioxidant activity. The MW (2 min) and MW (2 min) + US (10 min) extraction processes yielded the greatest betalain content in OS fresh weight (FW), with 48.54 ± 0.29 mg/100 g FW and 51.01 ± 0.16 mg/100 g FW, respectively. Furthermore, the results showed a considerable drop in betalain content when the plant material was dried at 40 °C and 60 °C, with reduction rates of 53.75% and 24.82%, respectively, compared to the betalain content before the drying process. The LC-DAD-ESI-MS analysis supported this result, revealing the presence of 17-decarboxy betanin, 17-decarboxy neobetanin, and Cyclo-dopa5-O-βglucoside in DOS at 40 °C. This study highlights the potential future in the sustainable green extraction of betalain compounds with less heat degradation to offer a stable natural colorant.

## 1. Introduction

In recent years, there has been a notable increase in consumer preference for natural and safe diets, which is attributed to the realization of the impact of food quality on personal health [1]. Unfortunately, the use of chemical additives in the food industry poses a significant challenge as they have been proven to be hazardous to both human and environmental health [2,3]. These additives have been linked to toxic and allergic reactions, hence resulting in an upsurge in demand for organic and natural food products [4,5]. Additionally, there has been a push for tighter regulations governing the use of chemical additives in the food industry. The increasing desire for organic and natural food products, coupled with more rigorous regulations concerning the incorporation of chemical additives within the food industry, has precipitated significant alterations. Amongst the various types of additives used in the food industry, colorants are the most used owing to their visual appeal, enhancement of the aesthetic value of food products, and even their impact on consumer behavior regarding purchasing. Consequently, natural pigments may seem like an attractive option for food manufacturers seeking to preserve colorant potential whilst avoiding the risks associated with synthetic products [6,7]. Moreover, natural colorants, derived from plant or animal sources, are perceived to be safer in terms of health [8,9]. Furthermore, this type of additive may provide nutritional benefits such as being a source of vitamins and antioxidant compounds [10,11,12].

The botanical species *Beta vulgaris* L., commonly known as red beetroot, is the most widely recognized source of natural food color owing to its betalain compounds. It is notable that Title 21 of the Code of Federal Regulations (21 CFR 73.40) permits its application as a red color additive in the United States, while the European Union identifies it as E162 [5,6]. The colorant is available in both liquid concentrate and spray-dried powder forms [13] and is primarily utilized to enhance the aesthetic appeal of food items, especially dairy products, confectioneries, tomato pastes, meat, and breakfast cereals [1,14,15,16]. In the past 10 years, other sources of betalains categorized under *Caryophyllales* have been identified. These sources are located in the vacuoles of various plant parts, namely seeds, fruits, roots, stems, and leaves, as reported by [17]. Notably, the *Amaranthaceae* family contains betalains in their roots, fruits, seeds, and bracts, such as *Beta vulgaris* L. [18,19,20], *Gomphrena globosa* L. [21], and *Amaranthus hypochondriacus* [22]. Additionally, the *Nycaginaceae* family possesses betalains in their flowers and bracts, such as *Bougainvillea glabra* flowers [23] and *Bougainvillea glabra* bracts [24], while Subfamily *Chenopodioideae* [25] and *Cactaceae* family (*Hylocereus* sp. [26], *Opuntia* spp. [27]) have betalains in their fruits.

The *Opuntia stricta*, a member of the *Cactaceae* family, is characterized by its acidic taste and striking red coloration. The extraction of coloring agent from this species could significantly enhance its value. The color range exhibited by this plant is attributed to the presence of betalains, a noteworthy group of secondary metabolic compounds. These pigments, which are nitrogen-containing, dissolve easily in water and are synthesized from betalamic acid. They are classified into two groups: betacyanin, which is red-violet in color and is a byproduct of the reaction between cyclo-DOPA and betalamic acid, and betaxanthin, which is yellow–orange and results from the interaction between betalamic acid and a variety of amines and amine acids [1,17,28].

Betalains are known to exhibit an acute sensitivity toward various environmental factors, including but not limited to temperature, pH, light, oxygen, water activity, metal ions, and enzymatic actions [17,29,30]. In particular, betalains are heat-sensitive molecules and degrade at temperatures above 60 ◦C [31]; this feature is a significant drawback for their use in industrial applications for food coloring [1]. Moreover, these molecules demonstrate a pH range spanning from 3 to 7, rendering them highly suitable for imparting color to low-acid and neutral foods. Conversely, anthocyanins, another widely employed group of natural colorants, which were extensively studied prior to the discovery of betalains in 1960, exhibit instability when exposed to pH levels exceeding 3, thereby limiting their application. Hence, the use of betalains pigments is deemed more favorable than anthocyanins for coloring foods that contain vitamin C [20,31].

The appearance of betalains is modulated by their intricate chemical structure. Betalains’ glycosylation or acylation structures and hydroxyl groups can enhance or diminish their capacity as free radical scavengers [17]. Recent research has shown that betacyanins display greater stability compared to bethaxanthins due to glycosylated structures with a high oxidation–reduction potential [32]. Moreover, other studies have demonstrated the remarkable potential of betalains as natural anti-inflammatory agents [33]. Betalains have also been found to possess antimicrobial properties, attributed to their chelating ability to trap cations such as Fe^2+^, Ca^2+^, and Mg^2+^ [17]. Given the significant health benefits conferred by betalains, their consumption has been correlated with a reduced risk of numerous diseases in humans [1,32,34].

Recently, there has been a focus on the retrieval of betalain compounds through novel and emerging technologies such as microwave-assisted extraction [24,35], ultrasound-assisted extraction [22,24,36,37], high pressure [38], and pulsed electric fields [39]. These methods are environmentally friendly and sustainable due to their ability to operate at low temperatures, under atmospheric pressure, and with minimal chemical input. They are also designed to reduce energy consumption and expedite processing demands, making them very effective in increasing betalain extraction yields from biological sources with minimal degradation [40,41,42,43].

In this study, an evaluation of microwave and ultrasound extraction technologies for betalains and polyphenols has been conducted. Although the mechanisms of both technologies widely vary, they share a commonality in the utilization of a cell disintegration process, which facilitates the extraction of bioactive compounds. Microwave technology employs electromagnetic radiation within the frequency range of 0.3 to 300 GHz, leading to water dipole rotation and ionic conduction that damages plant cells through internal heating, thereby releasing their contents into the solvent [41,44]. Conversely, ultrasound technology operates through wave propagation within the fluid containing the plant material, with frequencies between 20 and 100 kHz, resulting in the growth and violent collapse of bubbles near the plant material’s surface (cavitation). This occurrence alternately compresses and stretches the molecular structure, weakening the plant’s cell structure and allowing for solvent diffusion and content leaching [41].

Thus, the present study is conducted to investigate the influence of non- conventional extraction methods including both microwave and ultrasound technologies on bioactive compounds (betalains and polyphenols) of undried *Opuntia stricta* (OS) and dried *Opuntia stricta* (DOS) material. The objective was to develop an effective and environmentally friendly method for betalain extraction from *Opuntia stricta* plant.

## 2. Materials and Methods

### 2.1. Materials

The raw material *Opuntia stricta* var was harvested between February and March 2023 from the region of Sidi-Bouzid situated in the center of Tunisia. The average sizes of the fruit were as follows: pieces had a medium mass of 13.17 ± 3.23 g and a longueur and a larger of 3.94 ± 0.43 cm and 2.48 ± 0.27 cm, respectively. The fruits are characterized by a very thin peel with purple color.

Two kg of OS fruits (≈152 pieces) were carefully brushed to remove the glochids, washed, and ground in a mixer for 2 min without removing the peel and seeds. The mixture was then distributed in plastic bags and stored at −20 °C until experimental investigation. Three independent samples were analyzed for each accession and values reported are the mean ± standard deviation.

### 2.2. Drying Experiments

A part of the frozen material was dried in a convective dryer (Monferina EC25, Carmignano di Brenta, Italy) at 40 °C and 60 °C using a constant air velocity. In total, 100 g of thawed material was spread evenly in a thin layer with 2 mm thickness on a holder tray and placed inside the convective dryer. To investigate the temperature effect on bioactive molecule quality, drying kinetics were established by measuring the wet mass material (*M_t_*) variation at 20 min intervals during the drying process until reaching fixed mass of dried material [45]. The moisture content (*X_t_*: g water (g dry basis)^−1^) and the dimensionless moisture ratio (*MR*: (−)) were calculated according to (1) and (2), respectively [46]:

(1) $$X_{t}=\frac{M_{t}-M_{d}}{M_{d}},$$

where *M_t_* is the mass of wet sample material at instant t (g) and *M_d_* is the mass of dry sample material (g).

(2) $$MR_{t}=\frac{X_{t}-X_{eq}}{X_{0}-X_{eq}},$$

where *X_t_*, *X*_0_, and *X_eq_* refer to moisture content at time t, initial moisture content, and moisture content at equilibrium, respectively.

The initial moisture content of thawed material and the final moisture content of dried material were determined using a drying oven (Jouan, Saint Herblain, France) at 105 °C for 24 h. Drying experiments were performed until reaching equilibrium moisture content (*X_eq_*). This latter had previously been determined by the static gravimetric method for accurately approaching the drying process end. This method was based on the use of saturated salt solutions to maintain a fixed relative humidity in a closed space. The salts used were KOH, MgCl_2_, NaCl, KCl, and BaCl_2_, covering a relative humidity range of 5.5–87.3%. The saturated solutions were prepared by dissolving the required mass of salt in distilled water and then introduced inside hermetic glass jars. A mass of 1 ± 0.001 g of *Opuntia stricta* fruit was suspended at the jar’s lid, without encountering the solution, and then placed inside a drying oven at a controlled temperature. The thermodynamic equilibrium was considered to have been attained when a constant weight was reached [47]. After obtaining the desorption isotherms at 60 °C, the fruit sample was replaced, and the experiment was repeated at 40 °C.

Once drying was completed at 40 and 60 °C, the DOS was sifted to homogenate the particle size distribution. Then, it was kept in airtight containers until the extraction experiments.

### 2.3. Extraction Methods

The extraction of bioactive compounds (Betalains and TPC) was performed from OS and DOS material, using conventional and non-conventional methods (Microwave (MW), Ultrasound Bath (US), and combined microwave to Ultrasound Bath (MW/US)). The experimental parameters were adjusted referring to Castellar et al. [48] with little modifications. For all extraction methods, the water was used as solvent at a ratio of 1:10 and 1:50 (fruit:water), respectively, for OS and DOS reserving a constant dried mass since the moisture content of fresh material is about 80%. The homogenate was stirred for 20 min at a temperature of 50 °C for conventional extraction. The continuous stirring allows increasing the rate of extraction from the plant material. During the extraction process, the betalains were solubilized by the diffusion of the solvent in the plant material. The residual plant material was then separated from the liquid (extract) by applying centrifugation (Heraeus Biofuge Primo, Osterode, Germany) at 3000 rpm for 5 min and was reserved in falcon tubes for further analysis. These steps were performed quickly in the dark at room temperature to limit betalain degradation.

Non-conventional methods involved microwave (MW) or ultrasound (US) techniques, either alone or in combination. The duration of microwave and ultrasound application were chosen from the information gathered from the available bibliographical data. For the microwave extraction, the homogenate was introduced in a microwave oven (MS23K3513AK, Samsung, Suwon, Korea) at 300 W for 1 and 2 min noted, respectively, for MW (1 min) and MW (2 min). At the end of the treatment, the temperature of the homogenate did not exceed 50 ± 2 °C. For 1 min microwave treatment duration, the initial homogenate temperature was adjusted to attain a final temperature of 50 ± 2 °C. However, to maintain a constant final temperature of 50 °C for 2 min microwave treatment, an intermediate cooling was applied between two times 1 min extraction.

For the ultrasound extraction of bioactive compounds, the homogenate, reserved in a closed container, was introduced in an ultrasound bath (Branson 3510, Dambury, CT, USA) where the temperature is fixed at 50 °C and a frequency of 40 kHz for 10 and 20 min is noted, respectively, for US (10 min) and US (20 min).

For the combined microwave to ultrasound extraction, the homogenate underwent extraction with a microwave at 300 W for one or two min, with the temperature adjusted to not exceed 50 °C. Subsequently, the extraction was carried out using an ultrasound bath at 50 °C for 10 or 20 min. These combined methods were noted: MW (1 min) + US (10 min), MW (1 min) + US (20 min), MW (2 min) + US (10 min), and MW (2 min) + US (20 min). The different homogenates obtained with non-conventional methods were separated from the residual plant material by centrifugation at 3000 rpm for 5 min and were reserved in falcon tubes for further analysis. These steps were conducted in the dark at room temperature to limit betalain degradation. All the experiments were performed in triplicate.

### 2.4. Determination of the Total Betalain Content

The extracts from different conventional and non-conventional extraction methods were homogenized and then filtrated through a nylon filter (0.45 µm). The betalain content in extracts was evaluated using a UV–Vis spectrophotometer (Shimadzu, Kyoto, Japan). The absorbance of the extracts was measured at both 456 and 536 nm corresponding to the maximum absorbance of betaxanthin and betacyanin, respectively as deduced from the visible spectra (200–600 nm). The background absorption was measured at 600 nm for both of the pigments and the pigment contents were calculated according Equation (3). The results were expressed as mg betaxanthin or betacyanin per 100 g FW or 100 g DW [48].

(3) $$Betaxanthin\;or\;Betacyanin\;content=\frac{A\times MW\times V\times Df\times 1000}{\varepsilon \times i\times M},$$

where *A* = absorbance at 456 for betaxanthin and 536 nm for betacyanin, *MW* (molecular weight) = 308 g⋅mol^−1^ for betaxanthins and 550 g⋅mol^−1^ for betacyanins, *V* = total volume extract, *Df* = dilution factor, *ε* (molar extinction coefficient) = 48,000 L⋅mol^−1^⋅cm^−1^ for betaxanthin and 60,000 L⋅mol^−1^⋅cm^−1^ for betacyanin, *i* = path length of the cuvette (1 cm) and *M* = the fresh or dry weight of the plant material.

### 2.5. Determination of Total Polyphenol Content (TPC)

The Folin-Ciocalteu method was used to calculate the total polyphenol concentrations (TPC) [49]. For each sample 500 µL of the Folin-Ciocalteu reagent (10 times diluted) was mixed with 100 µL of appropriately diluted extract. After 5 min, 400 µL of a Na_2_CO_3_ (7.5%) solution were added. The absorbance was measured using an UV–Vis spectrophotometer (Shimadzu, Kyoto, Japan) at λ = 765 nm, after an incubation of 90 min in the dark. The polyphenol contents of the different extracts are expressed in mg of gallic acid equivalent per 100 g of fresh weight (g GAE/100 g FW) or dry weight (mg GAE/100 g DW).

### 2.6. Determination of Antioxidant Activity by 2,2′-Azino-Bis (3-Ethylbenzothiazoline-6-Sulphonic Acid) (ABTS) Assay

Antioxidant capacity of the extracts was measured using a protocol based on the ABTS assay [49]. The ABTS was dissolved in distilled water. ABTS radical cation (ABTS+) was produced by the reaction of ABTS stock solution with 2.4 mM potassium persulfate at the ratio of 1:1 and leaving the mixture to stand in the dark at 4 °C for 12–16 h before use. The resulting blue–green ABTS+ solution was diluted in ethanol to an absorbance of 0.791 ± 0.012 at 734 nm. An aliquot (10 μL) of each ethanolic extract was added to 990 µL of the resulting blue–green ABTS+. The mixture, protected from the light, was incubated for 20 min. The decrease in absorbance at 734 nm was measured by an UV–Vis spectrophotometer (Shimadzu, Kyoto, Japan). A Trolox (TE) standard calibration curve was elaborated to estimate the antioxidant capacity. The results were expressed as mmol TE equivalent per 100 g of fresh weight (mmol TE/100 g FW) or mmol TE equivalent per g of dry weight (mmol TE/g DW).

### 2.7. Chromatographic Analysis

For the identification of betalains, an LC (Alliance e2695) system was coupled to the MS 3100 single quadruple mass spectrometric technique (Waters, Milford, NZ, USA). A liquid chromatographic is equipped with pumps, an autosampler, and a Photodiode Array Detector PDA controlled with MassLynx software version 4.1 Samples were eluted through a 100 mm × 4.6 mm i.d., 5.0 µm, Waters C18 chromatographic column. The injection volume was 20 µL and the flow rate was 0.5 mL/min. The column was thermostated at 30 °C. Separation of the analytes was performed with a gradient system. The mobile phases were A—0.1% formic acid in water and B—0.1% formic acid in ACN. The gradient profile was (t (min); % A), (0; 95), (5; 95), (50; 0), (55; 0), (55.1; 95), and (60; 95).

The ionization electrospray source was operated in positive mode (ESI^+^). The capillary voltage was set at 3 kV, the cone at 30 V, and the source and the desolvatation temperature were 120 and 500 °C, respectively. Mass spectra and ion chromatograms were monitored in scan mode with *m*/*z* 200–1000.

The identification of betalains was based on a combination of retention times (RT), UV–Visible spectra, and a pseudomolecular ions, compared with previously reported data in the literature. The elution order followed typical chromatographic behavior, according to their polarity. The UV–Visible spectra showed characteristic maximum absorption wavelengths (λ max) for betacyanins in the range of 530–540 nm. Molecular ions were detected in positive ionization mode, confirming the expected pseudomolecular ions *m*/*z* for each betalain compound.

### 2.8. Statistical Analysis

All statistical analyses were conducted by means of the SPSS^®^ Statistics version 26.0 software for Windows (IBM, NewYork, NY, USA). The results were represented as mean ± standard deviation. Results obtained for the variables studied in the different groups were compared by one-way analysis of variance (ANOVA) with significant differences (*p*-value < 0.05) for normally distributed data and homogenized variances, by Welch for normally distributed data and non-homogenized variances, and with Kruskal–Wallis’s test for non-normally distributed data.

## 3. Results and Discussion

### 3.1. Extraction Method Effect on Betalains, TPC, and Antioxidant Activity in Opuntia stricta Fruit

From earlier work and reviews, experimental settings for both MW and US were adjusted given that the betalain extraction content (yield) is significantly influenced by temperature [40]. A temperature of 50 °C was recommended by several authors for bioactive compound extraction since it provides optimal yield extraction while preserving the molecules’ stability [1,43,50,51]. Specifically, betalains underwent degradation when exposed to temperatures above 60 °C [52]. Table 1 displays the betaxanthin, betacyanin, betalains, total phenolic content (TPC), and antioxidant activity for nine different extraction methods, including one conventional extraction method (Conv (20 min)) and eight non-conventional extraction methods, four (simple methods) of which used either microwave (MW (1 min) and MW (2 min)) or ultrasound bath ((US 10 min) and US (20 min)) and four (combined methods) with an association between microwave and ultrasound bath (MW (1 min) + US (10 min), MW (1 min) + US (20 min), MW (2 min) + US (10 min), and MW (2 min) + US (20 min)).

The results of betalain’s content ranged from 38.44 ± 0.97 to 51.01 ± 0.14 mg/100 g FW (Fresh Weight) depending on the extraction method used. The betacyanin, ranging from 37.24 ± 1.01 to 49.16 ± 0.10 mg/100 g FW, represented the main group at the rate of 96.70 ± 0.29%, which accounts for the strong purple hue of the OS plant. These results were in the same order as those reported in the literature [48,53,54,55] with 80.1 mg/100 g FW of Betacyanin using spectrophotometric analysis and 89.77 ± 4.49 mg/100 g FW quantified by HPLC-DAD-MS/QTOF. The high variability in betalain content reported is due to several factors, including ripeness, the part of the plant (peel, pulp) used for extraction, varieties, and whether the seeds are removed. Furthermore, the betalain level in the current study was within the range of the most prevalent betacyanin source found in beetroots (300–600 mg⋅kg^−1^), which is appreciated as a food colorant [44].

It was clearly demonstrated that non-conventional techniques, whether microwave or ultrasound and the combination of them, significantly increased the betalain content (*p*-value < 0.05). In fact, the highest betalain content was found with MW (2 min) + US (10 min), representing an increase of 18.52% over the conventional extraction method. Taking into account the duration of the extraction process, which varied depending on the methods used, the MW (2 min) + US (10 min) method thus allowed a 40% reduction in extraction time when compared to the conventional method (20 min). This extraction duration can be reduced by up to 90% with a MW (2 min) extraction method and a 13% increase in betalain content when compared to the conventional extraction method. Thus, the betalain content, using MW as the extraction method, was approximately 10–20 times higher when the extraction duration was considered. Cardoso-Ugarte et al. [56] confirmed this result where higher pigment percentages extracted from red beets using microwave-assisted extraction (MAE) are unregistered. According to this study, only 2–3 min of MAE are required to obtain an equivalent level of pigment extracted for 60 min under conventional extraction. This is mainly due to the vibration and friction of water molecules during microwave exposure, which results in a higher heating rate than traditional heating.

Moreover, the microwave effect at 300 W was more pronounced than the ultrasound (40 kHz) on betalain extraction since a significant difference was observed between 1 and 2 min microwave extraction duration with an increasing rate of about 19% versus 11% for the US extraction between 10 and 20 min. This result was similarly observed by Kuhn et al. [24] who compared different extraction methods of bioactive compounds from Bougainvillea glabra bracts. The best extraction performance was attributed to a microwave extraction recording 35–41% higher betalain yields than ultrasound and conventional aqueous extraction.

The results of increased extraction rates employing microwave and ultrasound technologies were confirmed using combined methods. In fact, they displayed a significant difference (*p*-value < 0.05) from the other MW or US methods. Nevertheless, the betalain rate was raised by 21.59% and 32.70% in MW (1 min) + US (10 min) and MW (2 min) + US (10 min), respectively, as compared to the ultrasound technique US (10 min). As the ultrasound time was set to 20 min, these rates were around 3.41% and 11.04% for MW (1 min) + US (20 min) and MW (2 min) + US (20 min), respectively. Thus, microwave technology outperformed ultrasound technology, even when used in tandem for betalain extraction.

Similar outcomes have been reported by Maran et al. [23] when ultrasound is used to extract pigments from Bougainvillea glabra flowers. Thus, the betalain extraction yield has been shown to exhibit an upward pattern up to 25 min before declining. Similarly, Laqui-Vilca et al. [25] also reported an increase in total betalain content, from colored quinoa (*Chenopodium quinoa Willd*) hulls, using ultrasound technology with the lengthening of the extraction period, until reaching a peak at about 10 s after which a negative effect is enregistered. More recently, Gómez-López et al. [36] showed a similar pattern for betalains and phenolic compounds extracted using ultrasonic from entire *Opuntia stricta var. dillenii* fruits. The most efficient extraction period was found to be 5 min, after which extraction yields decreased.

Compared to the conventional extraction method, non-conventional methods involving either MW, US technology, or combined MW/US significantly enhance the betalain content (*p*-value < 0.05) by the range of 2.97–18.52%. In fact, the MW rapidly transfers energy to the water content of the plant matrix causing internal heating in it, leading to the disruption of cell wall integrity. By adding the effect of US bath, the cavitation phenomenon acts directly on the internal parts of plant cells, such as the vacuoles that hold the pigments, resulting in a more pronounced effect on betalain extraction. This synergistic impact of combining environmentally friendly methods for bioactive compound extraction has already been noticed on various matrices besides prickly pears. In fact, it has been shown that associating ultrasonic-assisted extraction and microwave-assisted extraction led to the most effective recovery of bioactive components from date seeds without any significant difference in the order of both technologies [57]. Nevertheless, promising outcomes have been reported for the extraction of green coffee oil from Arabica coffee beans using a combined integrated ultrasonic-microwave technique in which microwave and ultrasound technologies are used simultaneously, compensating the effects of mechanical oscillation and ultrasound waves to the homogeneity of microwave heating [58]. However, this synergetic effect was not detected when combining high pressure and Ohmic heating techniques for extracting phenolic compounds from prickly pear peel [38].

Due to the lack of references available showing the association between different non-traditional technologies for betalain extraction from OS, this study contributes to improving betalain extraction. An increase in betalain content of 8.6–13% and 18% added to a significant decrease in extraction time of 40–55% and 90% for MW (2 min) + US (10 min), MW (1 min) + US (10 min), and MW (2 min), respectively, was reported. These non-conventional extraction techniques enable shorter extraction times and lower energy use, which may provide an intriguing solution for industry [35].

The total phenolic content (TPC) ranged from 1.82 ± 0.17 g GAE/100 g FW to 2 ± 0.25 g GAE/100 g FW, with no statistically significant differences among the extraction methods used in this study. These TPC values are higher than those published by Gómez-López et al. [55] with 104.58 ± 2.24 mg GAE/100 g FW who used frozen whole fruits *Opuntia stricta var dillenii* from the Canary Islands and Fernández-López et al. [54] who found 204.4 ± 4.2 mg GAE/100 g in *Opuntia stricta* fruit cultivated in Murcia (Spain). Aside from the variation in species localization and the extraction method, the solvent considered in the extraction process affects both the quantity and quality of molecules extracted. In fact, El Mannoubi [59] evaluated the solvent polarity effect on TPC from the OS plant. The unregistered TPCs were 16.349 ± 1.719 µg GAE/mg (DW) and 29.223 ± 1.894 µg GAE/mg (DW) in 80% ethanol and 80% acetone, respectively. This result was related to the lowest polarity of acetone, which enhances specifically the extraction of lipophilic polyphenols and flavonoids. It has also been shown that lower ethanol concentration (30% *v*/*v*) exerts a higher affinity for polyphenols in ultrasound extraction from beetroot waste [60]. Indeed, organic alcohol solvents were shown to extract fewer biomolecules, despite their role in reducing the coextraction of pectin, soluble fibers, and proteins interfering with betalain and polyphenols [60,61]. In this work, water was selected to favor the hydrophilic bioactive molecules extraction especially betalains beyond other hydrophilic soluble polyphenols as a safe natural alternative to some synthetic colorants maintaining an eco-friendly extraction process suitable for the food and pharmaceutical industry.

The different OS extracts are tested for their antioxidant properties using the ABTS free radical as scavenger radicals. The antioxidant activity, expressed as Trolox equivalent (TE), ranged from 97.44 ± 4.63 mmol TE/100 g FW to 128.78 ± 4.32 mmol TE/100 g FW. These values are greater than those reported by Betancourt et al. [62] for the fruit of the Colombian *Opuntia dillenii (Ker Gawl) Haw* cactus (35.24 ± 0.08 µmol TE/g fresh fruit) and Gómez-López et al. [36] for the Spanish variety of freeze-dried *Opuntia stricta var. Dilleni* (544.75 ± 14.37 µmol TE/g). The operating conditions in the extraction of bioactive molecules were the main cause for this large variation in antioxidant activity.

### 3.2. Extraction Method Effect on Betalains, TPC, and Antioxidant Activity in Dried Opuntia stricta Fruit

#### 3.2.1. Drying Kinetics

**Figure 1:**
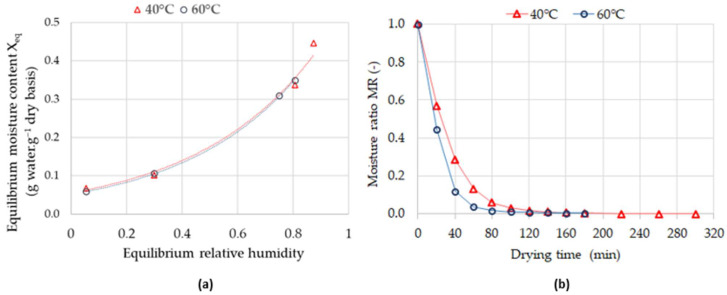
Desorption isotherms for *Opuntia stricta* fruit at 40 and 60 °C (**a**) Moisture ratio evolution versus drying time for *Opuntia stricta* fruit at 40 and 60 °C (**b**).

*The Opuntia stricta* plant tends to quickly deteriorate due to its high moisture content, which impacts the quality of bioactive compounds. To address this issue, the plant was dried at two different temperatures (40 and 60 °C) to evaluate the drying effect on the betalains, TPC, and antioxidant activity of OS material. The drying process was performed in a convective dryer at 40 and 60 °C. Starting with an initial OS moisture content of 76.367 ± 0.467%, the drying process was conducted until reaching a final moisture content of 8.662% ± 0.015 about 0.095 ± 0.0176 g water. (g dry basis)^−1^, determined by a mass variation of less than 0.01 g. This final moisture content is consistent with the equilibrium moisture content (*X_eq_*) of 0.0822 g water. (g dry basis)^−1^ and 0.0688 g water. (g dry basis)^−1^ at 40 and 60 °C, respectively, as determined from desorption curves (Figure 1a). Indeed, the drying process must be ended by reaching the equilibrium phase, which states that no more water can be removed by drying. In this case, the equilibrium moisture content was reached at 300 min and 180 min at 40 °C and 60 °C, respectively. The 66% increase in drying time when the temperature was reduced from 60 to 40 °C and may have an impact on the quality of bioactive compounds.

Figure 1b shows the moisture ratio of OS evolution versus drying time at 40 and 60 °C. The kinetic curves were quite similar for both temperatures with a drying occurring in the falling drying rate period. The period of constant drying rate was not observed, which is typically a common criterion of biological products. Moreover, as DOS material follows a falling drying rate, diffusion was identified as the main mechanism responsible for moisture transfer. This kinetic drying trend was similarly reported by Szadzińska et Mierzwa [46] for convective drying of white mushrooms and Gómez-Salazar et al. [63] for drying the peels of red prickly pear (*Opuntia streptacantha*).

#### 3.2.2. Drying and Extraction Method Effect on Bioactive Compounds

Table 2 shows the betalain content in OS and DOS extract using nine different extraction methods (one conventional and eight non-conventional methods). It was noticed that the OS quality material, either frozen or dried, significantly influenced the betalain content. The difference appeared to be more pronounced between OS and DOS material at 40 °C than between OS and DOS material at 60 °C. Indeed, the betalain content rate decreased up to 24.82% and 53.75% by drying at 60 and 40 °C, respectively. Thus, the drying temperature seems to significantly affect the betalain content. Indeed, at 40 °C, the OS was dried during 300 min versus 180 min at 60 °C to reach equilibrium moisture content. Thus, the couple 40 °C and 300 min affected more than 50% the betalain content. Similarly, Mella et al. [64] have reported the betalain degradation in beetroot undergoing vacuum drying at 40 °C. This phenomenon has been explained by the endogeneous enzyme’s activity (polyphenoloxidases (PPO) and peroxidases (POX)) at this specific temperature which resulted in higher betalain degradation.

Table 2 also presents the extraction method effect on betalain content. The higher betalain content was found for OS material with 215.83 ± 0.66 mg/100 g DW when associating microwave and ultrasound for extraction process (MW (2 min) + US (10 min)), followed by DOS material at 60 °C with 178.16 ± 7.68 mg/100 g DW with the US (20 min) and finally DOS at 40 °C with 122.03 ± 15.93 mg/100 g DW extracted by the non-conventional extraction method (MW (1 min) + US (10 min)). These findings demonstrated that the non-traditional extraction method (simple or combined) used in this study allowed for the extraction of increased betalain content when compared to the conventional extraction method by around 18%, promoting their use. Moreover, the extraction method effect on betalain content was different from what was previously observed with the betalain extraction from OS where microwave has shown better performance in comparison to the ultrasound technology (Section 3.1). Here, for DOS at 40 °C, by the increasing ultrasound or microwave duration, betalain content exhibited a significant decrease of 28.03%, 26%, and 17.45% for MW (1 min) to MW (2 min), US (10 min) to US (20 min), and MW (2 min) + US (10 min) to MW (2 min) + US (20 min), respectively. This might be due to the alteration that happened in the DOS matrice modifying its initial characteristic toward the extraction process. In fact, betacyanins are commonly known to be heat-eligible pigments [65]. So, long heating time may cause Betalain’s degradation by isomerization, decarboxylation, or cleavage, leading to a gradual decrease in red and the appearance of a light brown [1].

Table 3 reported the total polyphenol content for OS and DOS (40 and 60 °C) using nine different extraction methods (one conventional and eight non-conventional). The DOS material’s TPC ranged from 74.44 ± 3.10 mg GAE/g DW to 103.14 ± 7.94 mg GAE/g and from 61.17 ± 3.63 mg GAE/g DW to 75.53 ± 2.72 mg GAE/g DW at 40 °C and 60 °C drying temperatures, respectively. These values are lower than those obtained by Kharrat et al. [66] with 370.60 ± 0.12 µg GAE/mg of prickly pear extract from Tunisian *Opuntia stricta* variety (region Ghraba located in the North of Sfax) and considerably greater for another Tunisian *Opuntia stricta* variety collected from a botanical garden in Tunis with 0.75 mg GAE/g FW and 1.3 mg GAE/g FW for the pulp and peel, respectively [67]. In both these references, the authors used different drying processes such as drying at ambient temperature and freeze-drying, which modified the quality of the dried material.

Moreover, the highest TPC values were recorded for MW (1 min) (103.14 ± 7.94 mg GAE/g DW) and combined method (MW (2 min) + US (10 min)) (75.53 ± 2.72 mg GAE/g DW) for the DOS at 40 °C and 60 °C, respectively. These later data showed a TPC growth rate of 27.25% and 10.18% for the DOS at 40 °C and 60 °C, respectively, compared to the conventional extraction method. Therefore, regardless of the extraction method used, a substantially larger polyphenol content could be recovered at a lower drying temperature of 40 °C. This might likely be explained by the fact that polyphenols are more heat sensitive at 60 °C than at 40 °C, which is the opposite of what has previously been shown in the behavior of betalains. Similar results were reported by Gómez-Salazar et al. [63], who discovered that when the drying temperature increased, the levels of phenolic compounds declined while those of betalains increased. This tendency may be related to the production of indicaxanthin and betanin derivatives, which may raise pigment concentration. According to Ettalibi et al. [68], heat treatment between 50 and 60 °C can destroy phenolic structures, resulting in the generation of o-quinones and o-semi-quinones.

The TPC showed a statistically significant difference between OS and DOS material in four extraction methods (MW (1 min), MW (2 min), US (10 min), and MW (1 min) + US (10 min)).

Table 4 exhibited ABTS antioxidant activity in OS and DOS using nine different extraction methods (one conventional and eight non-conventional). The higher values of antioxidant activity were enregistered with non-conventional extraction methods as MW (2), MW (2 min) + US (10 min), and MW (2 min) + US (20 min) for OS and DOS (40, 60 °C), respectively. The antioxidant activity ranged from 4.17 ± 0.2 mmol TE/g DW to 5.51 ± 0.18 mmol TE/g DW, from 3.02 ± 0.15 mmol TE/g DW to 4.83 ± 0.21 mmol TE/g DW, and from 3.85 ± 0.18 mmol TE/g DW to 4.84 ± 0.20 mmol TE/g DW for OS and DOS at 40 °C and 60 °C, respectively.

The antioxidant activity of OS material is higher for all extraction methods tested, with the exception of MW (1 min) and MW (2 min) + US (10 min). In fact, prolonged exposure to high temperatures during the drying process can cause the formation of lower molecular weight compounds, which may lead to a decrease in the antioxidant capacities of the material. This finding has already been supported by Katanić et al. [69] who have shown that phenolics are not the primary driver of the antioxidant activity. In this study, it was reported a favorable connection between the betalain content and antioxidant activity against the ABTS radical (R = 0.405, *p*-value < 0.05) for the DOS material at 40 °C where the normal distribution was verified for Pearson correlation validity.

### 3.3. Betalain Identification by LC-DAD-ESI-MS

The qualitative determination of betalains was carried out using LC-DAD-ESI-MS for a selected extraction method (MW (2 min) + US (10 min)). In fact, betalain types, extracted from OS and DOS material (40 and 60 °C), could vary depending on the material’s quality. The peaks were confirmed by comparing mass spectra with those reported in the literature sources. For both OS and DOS material, the most intensive ion signal at 536 nm with [M+H]+ at *m*/*z* 551 correponded to Betanin (5-O-glucose Betanidin) [55,67,70]. Accordingly to previous studies, Betanin was the most abundant betalain compound in all *Opuntia* spp. and even in *Opuntia stricta* var. It was first isolated from red beetroot (*Beta vulgaris* L.) and approved as a Generally Recognized as Safe (GRAS) food additive. However, consumers’ demand for safe alternatives to synthetic dyes is growing. *Opuntia stricta* fruits can be used as potential sources of these natural pigments; therefore, it is important to know their betalain profile, their betalain content, and, especially, the optimal conditions for their extraction while preserving their biological activities.

For DOS (40 and 60 °C), other betanin derivatives were found including Betanidin [M+H]+ at *m*/*z* 389 [55,67,70], 17-decarboxy betanin with [M+H]+ at *m*/*z* 507 [55,70], 17-decarboxy neobetanin with [M+H]+ at *m*/*z* 505 [55], and Cyclo-dopa5-O-β glucoside with [M+H]+ at *m*/*z* 358 [55,70]. These fragments may be connected to the breaking of certain residues from the betanin structure, such as (*m*/*z* 162) for aglycone betanin (betanidin) or (*m*/*z* 44) for 17-decarboxy betanin. These findings could be justified by the thermal degradation of betalains to their derivatives. Table 5 summarizes the presence (+) or absence (−) of different betalain types identified in OS and DOS material; the majority of betalain derivatives were found in DOS (40 °C) material, where betalain underwent thermal degradation under a long drying process. This qualitative betalain identification confirmed the previous results in Section 3.2.2.

## 4. Conclusions

*Opuntia stricta* fruit, an underutilized widespread plant in Tunisia, was investigated for its potential as a natural source of betalain. Various extraction methods were applied, including the use of microwave and ultrasound technologies either alone or in combination. Both are regarded as eco-friendly techniques that are designed to increase the extraction of bioactive compounds while lowering energy use and expediting processing demands. As a result, the combined extraction method (MW (2 min) + US (10 min)) showed the greatest potential in terms of improving the betalain content by 18.52% and decreasing the extraction time by 40% in comparison to the conventional extraction method. In addition, the drying process was conducted to assess the stability of the betalain compound, revealing a decrease in the rate of betalain content in parallel with the drying time needed for this process. This drop rate is around 25% and 54 % at 60 °C and 40 °C drying temperatures, respectively. This result is also supported by LC-MS analysis where betalain derivatives (such as 17-decarboxy betanin, 17-decarboxy neobetanin, and Cyclo-dopa5-O-βglucoside) are mostly identified at 40 °C after 300 min of the drying process.

Overall, this study highlights the effectiveness of combining eco-friendly extraction methods for enhancing betalain content and other phenolic compounds in *Opuntia stricta* fruit. Nevertheless, great attention must be paid to the betalain degradation during thermal processes to preserve its stability. The *Opuntia stricta* fruit has proven potential as a natural colorant source in food applications.

## Figures and Tables

**Table 1:** Betaxanthin, betacyanin, betalains, total phenolic content TPC, and antioxidant activity for different extraction methods in OS material.

Methods	Betaxanthin mg/100 g FW	Betacyanin mg/100 g FW	Betalain mg/100 g FW	Total Phenolic Content g GAE/100 g FW	ABTS mmol TE/100 g FW
Conv (20 min)	1.27 ± 0.14 ^de^	41.77 ± 0.95 ^de^	43.04 ± 0.82 ^e^	2.00 ± 0.25	105.48 ± 3.02 ^d^
MW (1 min)	1.27 ± 0.09 ^de^	39.50 ± 1.02 ^f^	40.77 ± 1.12 ^f^	1.84 ± 0.04	113.78 ± 0.59 ^c^
MW (2 min)	1.60 ± 0.10 ^abcd^	46.94 ± 0.19 ^b^	48.54 ± 0.29 ^b^	1.89 ± 0.13	128.78 ± 4.32 ^a^
US (10 min)	1.21 ± 0.03 ^e^	37.24 ± 1.03 ^g^	38.44 ± 1.06 ^g^	1.87 ± 0.10	107.40 ± 3.02 ^d^
US (20 min)	1.66 ± 0.28 ^abc^	41.19 ± 0.46 ^e^	42.86 ± 0.73 ^e^	1.94 ± 0.33	110.72 ± 1.89 ^d^
MW (1 min) + US (10 min)	1.32 ± 0.23 ^cde^	45.42 ± 0.70 ^c^	46.74 ± 0.46 ^c^	1.97 ± 0.21	116.77 ± 4.50 ^bc^
MW (1 min) + US (20 min)	1.43 ± 0.16 ^bcde^	42.89 ± 0.29 ^d^	44.32 ± 0.14 ^d^	1.97 ± 0.09	120.00 ± 2.27 ^b^
MW (2 min) + US (10 min)	1.85 ± 0.06 ^a^	49.16 ± 0.10 ^a^	51.01 ± 0.16 ^a^	1.82 ± 0.17	97.44 ± 4.63 ^e^
MW (2 min) + US (20 min)	1.76 ± 0.30 ^ab^	45.83 ± 0.05 ^c^	47.59 ± 0.35 ^bc^	1.94 ± 0.28	119.29 ± 3.80 ^bc^
F-value	10.484	51.391	737.800	0.391	32.534
*p*-value	<0.05	0.000	0.000	0.916	0.000

All values given are means ± standard deviation (*n* = 6 Betalains and *n* = 4 TPC and ABTS). Means in columns with different letters are significantly different (*p*-value < 0.05).

**Table 2:** Betalain content in OS and DOS material (40 and 60 °C) expressed as mg Betanin equivalent per 100 g dry weight (DW).

Methods	Betalain mg/100 g DW OS	Betalain mg/100 g DW DOS at 40 °C	Betalain mg/100 g DW DOS at 60 °C	F-Value	*p*-Value
Conv (20 min)	182.12 ± 3.45 ^eA^	103.17 ± 6.08 ^bcC^	151.61 ± 5.50 ^dB^	15.445	0.000
MW (1 min)	172.51 ± 4.73 ^fA^	121.61 ± 8.42 ^aC^	147.93 ± 14.34 ^cdB^	15.494	0.000
MW (2 min)	205.38 ± 1.22 ^bA^	94.98 ± 9.63 ^cC^	169.21 ± 10.28 ^abcdB^	15.494	0.000
US (10 min)	162.67 ± 4.50 ^gA^	118.67 ± 3.53 ^aB^	162.56 ± 1.91 ^bcdA^	11.657	0.003
US (20 min)	190.22 ± 12.83 ^deA^	93.81 ± 12.76 ^cB^	178.16 ± 7.68 ^aA^	12.589	0.002
MW (1 min) + US (10 min)	197.79 ± 1.97 ^dA^	122.03 ± 15.93 ^abC^	174.82 ± 3.44 ^abB^	15.445	0.000
MW (1 min) + US (20 min)	187.53 ± 0.58 ^deA^	102.94 ± 0.75 ^bcC^	162.18 ± 10.70 ^abcdB^	15.477	0.000
MW (2 min) + US (10 min)	215.83 ± 0.66 ^aA^	121.47 ± 6.70 ^aC^	174.57 ± 9.67 ^abcB^	15.543	0.000
MW (2 min) + US (20 min)	201.38 ± 1.47 ^cA^	103.42 ± 9.40 b^bcC^	151.40 ± 18.83 ^cdB^	15.477	0.000
F-value	49.038	37.084	29.317		
*p*-value	0.000	0.000	0.000		

All values given are means ± standard deviation (*n* = 6), means in columns, and lines with different letters are significantly different (*p*-value <0.05).

**Table 3:** Total polyphenol content (TPC) in OS and DOS material (40 and 60 °C) expressed as mg gallic acid equivalent (GAE) per g DW.

Methods	TPC mg GAE/g DW OS	TPC mg GAE/g DW DOS at 40 °C	TPC mg GAE/g DW DOS at 60 °C	F-Value	*p*-Value
Conv (20 min)	84.69 ± 10.53 ^A^	81.05 ± 7.07 ^abA^	68.55 ± 9.9 ^abA^	3.311	0.084
MW (1 min)	78.01 ± 1.56 ^A^	103.14 ± 7.94 ^aB^	61.61 ± 16.36 ^abA^	15.757	0.001
MW (2 min)	80.21 ± 6.59 ^AB^	89.88 ± 13.46 ^abA^	68.40 ± 4.00 ^abB^	5.774	0.024
US (10 min)	79.22 ± 4.12 ^A^	74.44 ± 3.10 ^bA^	63.28 ± 7.57 ^abB^	9.581	0.006
US (20 min)	82.33 ± 13.99 ^A^	84.77 ± 16.29 ^abA^	73.11 ± 11.42 ^abA^	0.612	0.585
MW (1 min) + US (10 min)	83.39 ± 8.78 ^A^	76.12 ± 5.90 ^abA^	61.17 ± 3.63 ^bB^	12.307	0.003
MW (1 min) + US (20 min)	83.58 ± 3.96 ^A^	76.41 ± 15.60 ^bA^	75.39 ± 8.11 ^abA^	1.666	0.28
MW (2 min) + US (10 min)	76.95 ± 7.37 ^A^	79.90 ± 11.84 ^abA^	75.53 ± 2.72 ^aA^	0.268	0.776
MW (2 min) + US (20 min)	81.96 ± 12.07 ^A^	87.28 ± 7.08 b^abA^	73.30 ± 18.47 ^abA^	1.003	0.427
F-Value	0.614	4.855	4.143		
*p*-Value	0.758	0.009	0.016		

All values given are means ± standard deviation (*n* = 4), means in columns, and lines with different letters are significantly different (*p*-value <0.05).

**Table 4:** ABTS antioxidant activity in OS and DOS material (40 and 60 °C) expressed as mmol Trolox equivalent (TE) per g DW.

Methods	ABTS mmol TE/g DW OS	ABTS mmol TE/g DW DOS at 40 °C	ABTS mmol TE/g DW DOS at 60 °C	F-Value	*p*-Value
Conv (20 min)	4.51 ± 0.13 ^dA^	4.10 ± 0.25 ^abAB^	4.01 ± 0.06 ^bcB^	20.269	0.004
MW (1 min)	4.87 ± 0.03 ^cA^	4.44 ± 0.49 ^abA^	4.45 ± 0.15 ^abA^	5.753	0.056
MW (2 min)	5.51 ± 0.18 ^aA^	3.61 ± 0.13 ^bcB^	4.33 ± 0.33 ^abcB^	125.834	0.000
US (10 min)	4.60 ± 0.13 ^dA^	3.85 ± 0.18 ^abcB^	4.07 ± 0.11 ^abcB^	26.969	0.000
US (20 min)	4.74 ± 0.08 ^dA^	4.09 ± 0.35 ^abcAB^	4.45 ± 0.19 ^abcB^	7.225	0.013
MW (1 min) + US (10 min)	5.00 ± 0.19 ^bcA^	4.05 ± 0.73 ^abcAB^	3.85 ± 0.18 ^cB^	29.818	0.001
MW (1 min) + US (20 min)	5.14 ± 0.10 ^bA^	3.03 ± 0.86 ^bcB^	4.77 ± 0.67 ^abcA^	7.463	0.024
MW (2 min) + US (10 min)	4.17 ± 0.20 ^eB^	4.83 ± 0.21 ^aA^	4.05 ± 0.09 ^bcB^	22.501	0.000
MW (2 min) + US (20 min)	5.11 ± 0.16 ^bcA^	3.02 ± 0.15 ^cB^	4.84 ± 0.20 ^aA^	8.085	0.018
F-value	32.534	24.008	21.073		
*p*-value	0.000	0.002	0.007		

All values given are means ± standard deviation (*n* = 4), means in columns, and lines with different letters are significantly different (*p*-value < 0.05).

**Table 5:** The betalain derivatives identification in different material OS, DOS at 40 °C, and DOS at 60 °C. (+) presence of the compound, (−) absence of the compound.

Betalain	[M+H]+	OS	DOS 40 °C	DOS 60 °C
Betanin	551	+	+	+
Betanidin	389	+	+	−
17-decarboxy betanin	507	−	+	+
17-decarboxy neobetanin	505	−	+	−
Cyclo-dopa5-O-βglucoside	358	−	+	−

## Data Availability

The original contributions presented in the study are included in the article, further inquiries can be directed to the corresponding author.
